# Intraoperative Ultrasound: An Old but Ever New Technology for a More Personalized Approach to Brain Tumor Surgery

**DOI:** 10.7759/cureus.62278

**Published:** 2024-06-12

**Authors:** Gervith Reyes Soto, Carlos Murillo Ponce, Carlos Catillo-Rangel, Bernardo Cacho Diaz, Renat Nurmukhametov, Gennady Chmutin, Jeff Natalaja Mukengeshay, Cherubain Mpoyi Tshiunza, Manuel de Jesus Encarnacion Ramirez, Nicola Montemurro

**Affiliations:** 1 Neurosurgical Oncology, Instituto Nacional de Cancerología, Mexico City, MEX; 2 Head and Neck Surgery, Instituto Nacional de Cancerología, Mexico City, MEX; 3 Neurosurgery, Hospital Regional 1ro de Octubre (ISSSTE or Instituto de Seguridad y Servicios Sociales de los Trabajadores del Estado), Mexico City, MEX; 4 Neurosurgery, 2nd National Clinical Centre of Federal State Budgetary Research Institution (Russian Research Center of Surgery named after Academician B.V. Petrovsky), Moscow, RUS; 5 Neurosurgery, Peoples' Friendship University of Russia (RUDN University), Moscow, RUS; 6 Neurosurgery, Clinique Ngaliema, Kinshasa, COD; 7 Neurosurgery, Azienda Ospedaliera Universitaria Pisana (AOUP) University of Pisa, Pisa, ITA

**Keywords:** surgical procedure, neurosurgery, metastases, glioma, intraoperative ultrasound, glioblastoma

## Abstract

Background: Although the use of transcranial ultrasound dates to the mid-20th century, the main purpose of this research work is to standardize its use in the resection of brain tumors. This is due to its wide availability, low cost, lack of contraindications, and absence of harmful effects for the patient and medical staff, along with the possibility of real-time verification of the complete resection of tumor lesions and minimization of vascular injuries or damage to adjacent structures.

Methods: A retrospective study was conducted from June to December 2022. The study included eight patients (three men and five women) aged between 32 and 76 years. Histological examination revealed two high-grade gliomas, one low-grade glioma, and five metastatic lesions.

Results: The low-grade glioma appeared as a homogeneously echogenic structure and easily distinguishable from brain parenchyma, whereas metastases and high-grade gliomas showed higher echogenicity, being identified as malignant lesions due to areas of low echogenicity necrosis and peritumoral edema identified as a hyperechogenic structure.

Conclusions: The use of intraoperative transcranial ultrasound constitutes an important tool for neurosurgeons during tumor resection. Although it is easy to use, intraoperative ultrasound requires a relatively short learning curve and a good understanding of the fundamentals of ultrasound. Its main advantage over neuronavigation is that it is not affected by the "brain shift" phenomenon that commonly occurs during tumor resection, since the ultrasound images are updated during surgery.

## Introduction

Globally, brain metastases are the most common brain tumors, occurring in approximately 40% of all cancer patients during their illness [1,2]. The primary goal of treating all patients with primary tumors and brain metastases is maximum surgical resection, with local control of their disease and improvement in their quality of life. This is the major determinant in terms of survival and is closely related to the prognosis of their illness. Therefore, in recent decades, intraoperative imaging techniques have been developed that help to perform a complete and safe surgical resection of these lesions effectively [1-3]. The use of intraoperative ultrasound in neurosurgery dates back to the 1980s. However, its use was limited by the poor quality of images at that time. However, with new technological advances, it is now possible to obtain better real-time images that have advantages over preoperatively derived images [4]. This is because structures move during surgery and new characteristics can develop, such as hydrocephalus or hemorrhage. Although its quality does not surpass that of magnetic resonance imaging (MRI), this intraoperative tool is not biased by brain shift and is ideal for instantly locating and visualizing intracerebral lesions during surgery. It is also relatively inexpensive and easy to use and requires little intraoperative equipment or maintenance [5]. This evolution of intraoperative ultrasound in neurosurgery represents a significant leap in the field of neurooncology [6]. The enhanced imaging capability allows surgeons to distinguish between tumor tissue and normal brain tissue more effectively, increasing the precision of tumor resections [7]. This precision is especially crucial in brain surgery, where the margin for error is minimal and the preservation of healthy brain tissue is vital for maintaining the patient's cognitive and functional abilities [8]. Furthermore, the real-time aspect of intraoperative ultrasound provides an unparalleled advantage. It enables neurosurgeons to adjust their surgical strategies dynamically as the surgery progresses. This adaptability is particularly important in cases where brain shift can alter the landscape of the surgical field. Traditional imaging methods, like preoperative MRI, cannot account for these intraoperative changes, making intraoperative ultrasound a more reliable guide [9,10].

The integration of intraoperative ultrasound into neurosurgical procedures also signifies an advancement in patient safety. By facilitating more accurate tumor resections, it potentially reduces the need for repeat surgeries, which carry additional risks and burdens for patients [11]. Additionally, the non-invasiveness and absence of ionizing radiation in ultrasound technology make it a safer imaging alternative, especially for patients who may have contraindications to MRI, such as those with pacemakers or other metallic implants [12]. The cost-effectiveness of intraoperative ultrasound is another factor that cannot be overlooked. In resource-limited settings, where access to high-end imaging technologies like intraoperative MRI is scarce, intraoperative ultrasound stands out as a viable, less expensive alternative [12,13]. It provides neurosurgeons with the essential imaging support needed to perform complex brain surgeries without the substantial financial investment required for more advanced imaging technologies [14]. Looking ahead, the continuous improvement of ultrasound technology, including enhancements in image resolution and the integration of 3D imaging, promises to further revolutionize its application in neurosurgery [15]. These advancements are likely to expand the capabilities of neurosurgeons, allowing for even more precise and safe surgical interventions and improving the outcomes and quality of life for patients with brain tumors [16].

Despite the use of new emerging technologies such as augmented reality, mixed reality, and exoscope [17-19], intraoperative ultrasound is becoming progressively more common during brain tumor surgery [20], benefiting a wider range of patients across the world, particularly in low-income countries [21]. The purpose of this paper is to determine the ultrasonographic characteristics of different intracerebral tumoral and metastatic lesions, their relationship with adjacent brain tissue, their vasculature, and the degree of resection in real time in order to improve the use of this technique.

## Materials and methods

Study design

This descriptive retrospective study focuses on evaluating the efficacy and outcomes of intraoperative ultrasonography in brain tumor surgeries, specifically targeting primary brain tumors and metastatic lesions.

The population for this study comprised patients diagnosed with primary brain tumors or brain metastases who underwent surgical resection at Instituto Nacional de Cancerología, Mexico City, from June to December 2022. The use of intraoperative ultrasonography was a common factor in all these cases. Data were analyzed using appropriate statistical techniques to assess the impact of intraoperative ultrasound on surgical outcomes, including the extent of tumor resection and the occurrence of intraoperative adjustments or postoperative complications.

Inclusion and exclusion criteria

Patients with a confirmed diagnosis of primary or secondary brain tumor who underwent surgical resection with intraoperative ultrasound from June to December 2022 were included in this study. In contrast, patients with uncompleted intraoperative ultrasonographic study (two out 10) due to technical difficulties or unavailability were excluded.

Data collection and management

Data were collected retrospectively from patient medical records. This included demographic information, tumor type and location, details of the surgical procedure, intraoperative ultrasound findings, and postoperative outcomes. The data collection tools were designed to ensure the comprehensive capture of relevant information while maintaining the confidentiality of patient identities.

Ultrasonography technique

An intraoperative ultrasound C42T probe (miniconvex, 1-13 MHz, 65°, Hitachi Aloka ProSound Alpha 7 Medical Ltd., Japan) for neurosurgical applications was used. The objective was to evaluate the accuracy of tumor localization, the clarity of tumor margins, and the effectiveness in differentiating tumor tissue from healthy brain tissue.

Ethical considerations

The research protocol was developed in accordance with national and international ethical guidelines for health research. As a retrospective study, it was classified as "no-risk" research. The confidentiality of patient data was a top priority, with stringent measures taken to anonymize and secure all information. The risk-benefit balance was thoroughly considered, emphasizing the potential broader benefits of this research in enhancing neurosurgical practices and patient outcomes. Approval was obtained from the Ethics Committee of Instituto Nacional de Cancerología (approval number: 01/2019).

## Results

A descriptive analysis was carried out, allowing for the identification of frequency variations in each of the variables of the study. The data analysis was processed using IBM SPSS Statistics for Windows, Version 25.0 (Released 2017; IBM Corp., Armonk, New York, United States). Categorical variables were presented in both frequency (n) and percentages (%). The study included eight patients, three men and five women, with ages ranging from 32 to 76 years (average age: 55.3 years old). There were three frontal craniotomies, two parietal, one frontoparietal, one frontotemporal, and one occipital. In five patients, the tumor was located in the left cerebral hemisphere, two in the right cerebral hemisphere, and one at the right cerebellar paravermian level. The histological study revealed two high-grade gliomas, one low-grade glioma, and five metastatic lesions. The demographic characteristics of the patients studied, as well as the type of primary or metastatic lesion, tumor location, and preoperative and postoperative neurological status, are shown in Table 1.

**Table 1:** Demographic characteristics of the patients included in this study M: male; F: female; LGG: low-grade glioma; GBM: glioblastoma

Case no.	Sex/age	Location	Preoperative neurological status	Histopathological diagnosis	Extent of resection	Postoperative neurological status
1	F/57	Right frontoparietal	Left hemiparesis	Metastasis (ovarian adenocarcinoma)	Total resection	Improvement of hemiparesis
2	F/59	Right frontal (precentral gyrus)	Left hemiparesis	Metastasis (melanoma)	Total resection	Improvement of hemiparesis
3	F/76	Left frontal	Conduction aphasia	Metastasis (clear cell renal carcinoma)	Total resection	Improvement of aphasia
4	F/32	Right paravermian	None	Metastasis (invasive ductal carcinoma of the breast)	Total resection	No changes
5	M/54	Left frontal	Right hemiparesis with motor aphasia	LGG	Supratotal resection	No changes
6	M/38	Left frontotemporal	None	LGG	Supratotal resection	Intermittent dysnomia
7	M/73	Left parietal	Right hemiparesis	GBM	Supratotal resection	Improvement of hemiparesis
8	F/54	Left parietal	Right hemiparesis	Metastasis (osteosarcoma)	Total resection	Improvement of hemiparesis

**Figure 1:**
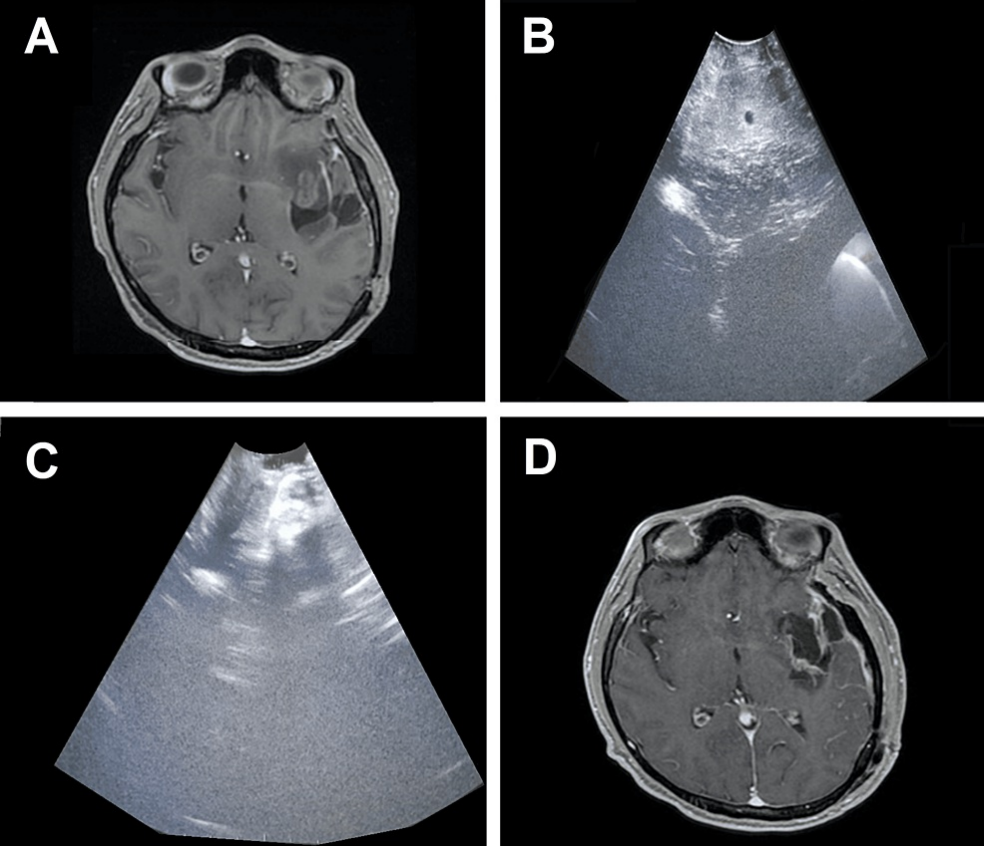
(A) Preoperative magnetic resonance imaging in which left frontotemporal (insular) low-grade glioma is observed. (B) Intraoperative ultrasonography image after craniotomy where homogeneous echogenic image is observed that differs from the normal but hypoechogenic brain. (C) Post-resection image of the tumor where a radical resection is observed. (D) Postoperative magnetic resonance imaging in which the degree of tumor resection is corroborated

**Figure 2:**
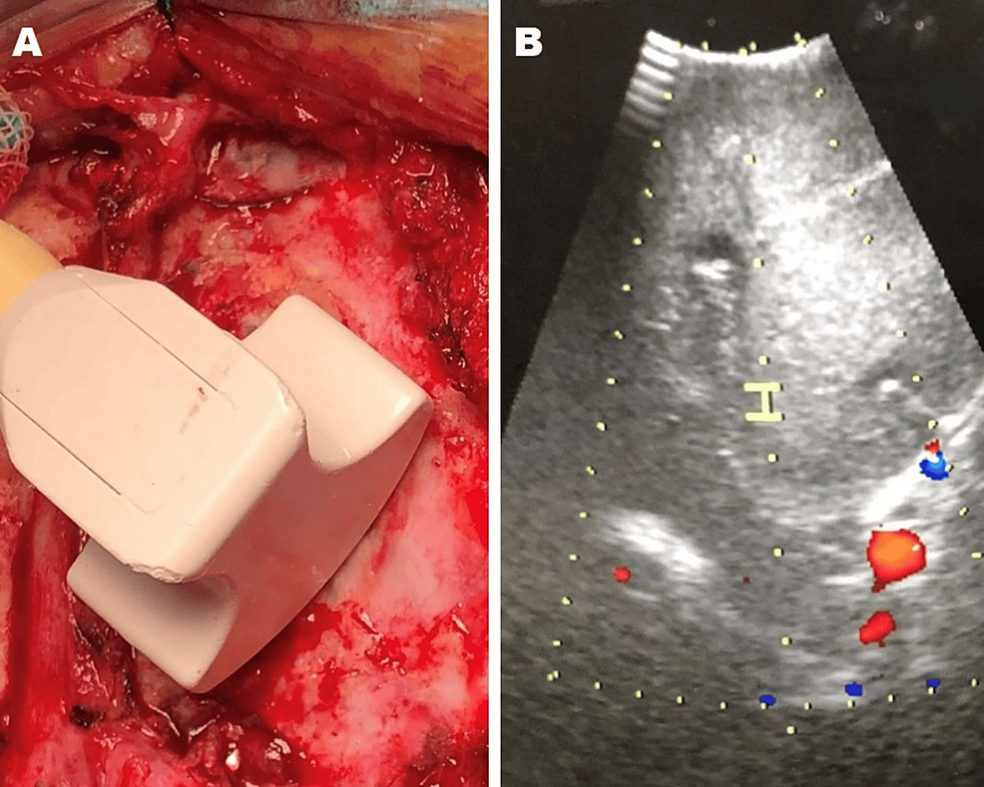
A 76-year-old patient with left frontal metastasis of clear cell renal carcinoma. (A) Placement of the transducer in the intraoperative, after having performed the craniotomy. (B) Intraoperative ultrasonography image after craniotomy where heterogeneous hyperechogenic intra-axial image is observed; tumor vessels are also observed

**Figure 3:**
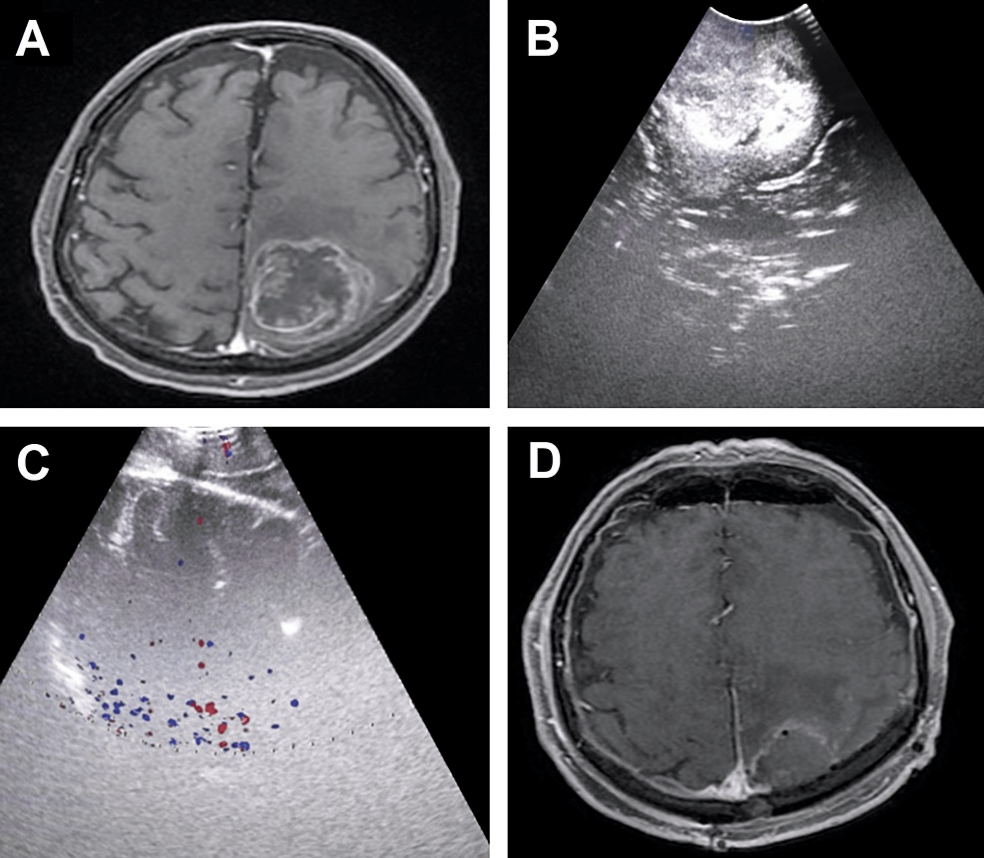
(A) Preoperative magnetic resonance imaging of a 73-year-old patient with a left parietal glioblastoma with perilesional edema. (B) Intraoperative ultrasonography image prior to dural opening where heterogeneous hyperechogenic image is observed, with hypoechogenic zones corresponding to necrosis. (C) Trans-surgical ultrasound image post-resection of the tumor where a radical resection is observed, without evidence of residual injury or bleeding. (D) Postoperative magnetic resonance imaging in which the degree of tumor resection is corroborated

The low-grade glioma was shown as homogeneous echogenic structures, easily distinguishable from brain parenchyma (Figure 1). However, metastases and high-grade gliomas (Figure 2 and Figure 3) showed greater echogenicity than low-grade gliomas, being identified as malignant lesions due to areas of low echogenicity necrosis and peritumoral edema identified as a hyperechogenic structure. No complications were recorded during tumor excision in any of the patients in relation to the technique, nor were hemorrhagic complications or added neurological deficits detected. Also, no tumor remnants were detected at the end of the surgery. The performance of a postoperative cranial MRI confirmed the total excision of the tumor in the rest of the patients studied.

## Discussion

This study demonstrates that intraoperative ultrasound is useful for locating supra- and infratentorial intra-axial brain tumors, as well as for detecting tumor remnants or bleeding after tumor excision. In line with this and agreeing with other authors, in our series of eight patients, we located the tumor after the craniotomy and before the dural opening, with high precision regardless of the histopathological diagnosis. Similarly, it was possible to identify the tumor borders before resection in all patients included in this study [5,22]. We have also found that intraoperative ultrasound allows for the detection of the demarcation line between the tumor and the brain, noting that the borders of high-grade gliomas and metastases are denser and more irregular than in low-grade gliomas, due to the invasive nature of malignant tumors [23,24]. Additionally, the necrotic component is primarily observed in high-grade gliomas and metastases, appearing sonographically as low echogenicity, located in the central and paracentral area of the tumor parenchyma. On the other hand, it has been noted that cystic lesions appear as hypoechoic areas surrounded by solid tumor tissue or membranous structures [24,25].

Various authors have demonstrated in patients with brain tumors an increase in survival time related to the degree of tumor resection [26,27]. Moreover, recent publications have shown that intraoperative ultrasound provides better information about the extent and location of the tumor, facilitating the neurosurgeon's orientation during its excision [28,29]. Intraoperative ultrasound also aims to complement neuronavigation images, whose validity decreases when there is a displacement of brain tissue during tumor resection, a phenomenon known as "brain shift" [24]. This limitation of neuronavigation occurs particularly in supratentorial tumors and even more so if they have a cystic component. Therefore, intraoperative ultrasound, being an imaging study obtained in real time, is updated with each new control, delivering precise anatomical information, not affected by any displacement of brain tissue that may occur during surgery. Each ultrasonographic evaluation should take only minutes to perform and therefore should not delay the usual surgical times.

Detecting a possible tumor remnant during surgery can mean significant time and cost savings for the patient, avoiding a possible surgical reintervention [28-32]. Finally, intraoperative ultrasound is a technology that is less costly compared to other studies such as intraoperative MRI. Additionally, this technology is safe as it does not use ionizing radiation and the incidence of other possible types of complications such as infection or brain damage due to compression is no higher than that of the surgical intervention itself [30]. Intraoperative orientation in neurosurgery remains a crucial step. Anatomical topographic reference points, neuronavigation, and the advent of intraoperative imaging techniques such as ultrasound, tomography, and MRI have enabled neurosurgeons in recent years to locate lesions and their surrounding structures, help the neurosurgical approach, and achieve a safe maximal resection [1,4,6]. The alternatives for intraoperative monitoring in neurosurgery and more specifically in neurooncology have increased over the past decades, especially compared to the pre-magnetic resonance era [7,8]. This monitoring aims to improve surgical safety, limiting the morbidity of tumor resection as also optimizing the results of tumor resection. Intraoperative monitoring has incorporated multiple modalities including neuroimaging, neurophysiological monitoring, and functional clinical monitoring of awake surgery [14]. Available neuroimaging options include neuronavigation techniques using magnetic resonance or computed tomography images, optical techniques such as tumor fluorescence, conventional radiology, intraoperative tomography and MRI, and intraoperative ultrasonography [9,10,15].

Although much has been written about intraoperative MRI, the enormous costs involved and the relative impracticality indicate that it is likely to remain out of reach for many neurosurgery units worldwide in the medium term. In glioma surgery, methods such as intraoperative computed tomography (ICT), intraoperative magnetic resonance imaging (IMRI), navigation, 5-aminolevulinic acid (5-ALA), and intraoperative ultrasound are used to achieve an expanded resection during the surgical procedure [29]. Intraoperative ultrasound has been increasingly used in the surgery of high-grade gliomas and various tumors due to its convenient intraoperative use, its flexible repeatability, and the relatively low cost of operating room construction [29]. Therefore, the objective of this study is to focus on the use of intraoperative ultrasound and the characterization of brain lesions, which, although its use was limited in past decades, is now a completely renewed technique with a notable improvement in image acquisition, thus regaining attention in the field of neurosurgery [11-13]. Intraoperative ultrasound is used in different neurosurgical subspecialties. The main applications are as follows: neurooncology (tumor localization, degree of resection evaluation, identification of draining vessels in vascularized tumors, and evaluation of venous sinus permeability) [14], infection (localization, identification, and ultrasound-guided aspiration), vascular (localization and identification of vascular lesions and feeding and draining vessels) [14,33,34], and pediatrics (facilitating transfontanellar ultrasound exploration of the intracranial space for a quick macroscopic evaluation of possible existing lesions, this window also helps assess hydrocephalus or the correct positioning of the ventricular catheter, both intraoperatively and postoperatively, and can complement existing image-guided techniques with real-time imaging [14-17]).

Indications in neurooncological surgery

Localization of the Lesion and Planning of the Optimal Approach

**Figure 4:**
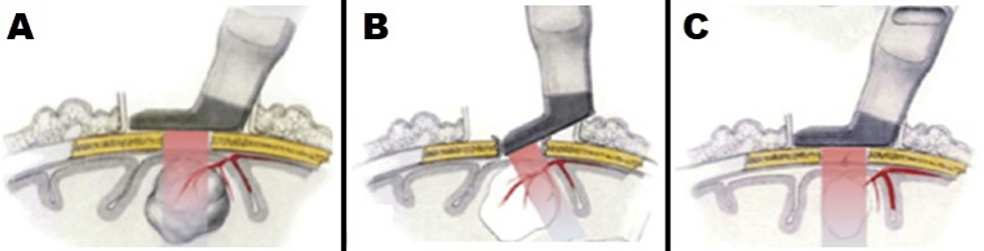
Graphical representation of the use of intraoperative ultrasound in the resection of a brain tumor. (A) The transducer is placed over the craniotomy and lesion; (B) tumor vascularization; (C) control after tumor resection Reference: [1]

Intraoperative ultrasound is indicated in any supra- and infratentorial intra-axial brain tumor. Ultrasound images can differentiate normal versus pathological tissue 80-88% of the time, and their use increases tumor resection by 55%, particularly if the tumor has heterogeneous tissue with cystic components [35,36]. Additionally, intraoperative ultrasound allows for better visualization of the surgical access to the lesion, providing information on distance, size, and interposition or proximity to major vessels or ventricles. Small subcortical lesions (especially cavernomas, metastases, and hemangioblastomas) are easily visible [37,38] (Figure 4A).

Vascular Structures

In tumor surgery, locating the main arteries and veins in relation to a tumor, including the permeability of venous sinuses, as well as assessing tumor vascularization helps ensure a safe resection [27,37-40] (Figure 4B).

Resection Control

Lesions with clearly visible margins before resection (most metastases, meningiomas, and some gliomas) can be used to verify if the resection is complete. The fusion of intraoperative ultrasound images with neuronavigation is particularly useful for the intraoperative orientation and assessment of tumor resection, and then, after tumor resection, it is possible to evaluate the presence of possible residual tumor in the surgical bed [41-43] (Figure 4C).

Postoperative Complications

After dural closure and immediately before the replacement of the bone flap, a final review with ultrasound allows for the identification of an early intracerebral hematoma or hydrocephalus [44,45].

General principles of ultrasound

For a better understanding of the physics of ultrasound, some definitions should be specified. Ultrasound, as its name suggests, is a high-frequency sound. It is generated by a transducer that converts electrical signals into ultrasound waves, collects the reflected signals, and reconverts them into electrical signals. These signals are displayed on the screen [28,46]. Attenuation describes the loss of energy, expressed as a change in intensity, as sound waves travel through a medium. Ultrasound is reflected at the boundaries between different materials.

In neurosurgery, this means that if the consistency between two different tissues (e.g., normal brain and tumor) is significantly different, the lesion will be clearly visible. However, if the tumor tissue has a consistency similar to the normal brain (as in some low-grade gliomas), it is more difficult to distinguish the difference [46,47]. The ultrasound image is formed by a matrix of photographic elements called pixels varying in brightness represented in grayscale according to the proportion of echo intensity. The brightness intensity of the ultrasound image is represented on a grayscale and is called "echogenicity" [46-48].

Characteristics of the image

Characteristics of normal structures are as follows [48]: (a) hyperechogenic structures (bright on intraoperative ultrasound) include the falx, tentorium, choroid plexus, and pineal gland; (b) isoechogenic structures (isointense on intraoperative ultrasound) include the normal brain tissue; (c) hypoechoic structures (dark on intraoperative ultrasound) include the brainstem; and (d) anechoic structures (without signal) include the ventricles and basal cisterns.

**Figure 5:**
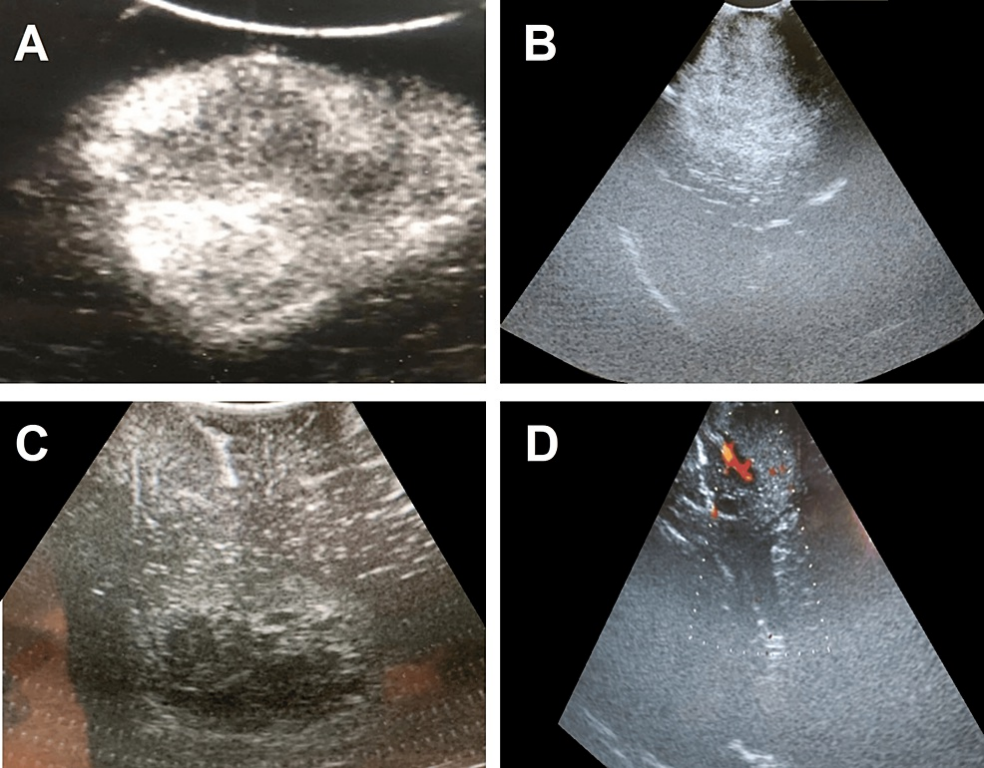
Intraoperative ultrasound images of tumor lesions from patients included in our study. (A) Brain metastasis from breast carcinoma; (B and C) high-grade gliomas; (D) vascularization in a tumor detected using ultrasound

Pathological structures have heterogeneous echogenicity with areas of hypo- and hyperechogenicity. Hyperechoic lesions include fresh blood, most metastatic lesions, meningiomas, the solid part of craniopharyngiomas, hemangiomas, the capsule of cystic lesions, calcification, and some gliomas. Moderately hyperechoic lesions include most glial tumors, edema, and some metastases. Hypoechoic or anechoic lesions include most cystic lesions, old hematomas, and the necrotic part of a glioblastoma [47-49] (Figure 5).

Limitations of the study

This study has some limitations. First, the sample size was small. A larger sample size would provide statistical analysis and enhance the generalizability of the findings. In addition, this study has no follow-up. Furthermore, the absence of a control group who underwent surgery without ultrasound limits the ability to directly compare the treatment groups.

## Conclusions

The use of intraoperative brain ultrasound constitutes an important tool for neurosurgeons during tumor resection. It is easy to use and requires a relatively short learning curve and a good understanding of the fundamentals of ultrasonography to accurately obtain and interpret images. Its main advantage over neuronavigation is that it is not affected by the "brain shift" phenomenon that usually occurs during tumor resection, as the ultrasound images are updated during surgery. Based on our clinical experience, intraoperative ultrasonography provides an acceptably high-quality image as a complement for the resection of supra- and infratentorial intra-axial brain tumors, as it allows for their reliable and safe localization, especially in tumors located in areas of the brain with significant anatomical and functional importance, as well as to identify tumor remnants once the surgery is completed. Additionally, intraoperative ultrasound is a cost-effective, quick, and easy-to-handle method for neurosurgeons. For these reasons, the authors of this manuscript recommend ultrasonography as a routine procedure for the excision of brain tumors.
